# Dietary Risk Among Older Adults: Examining the Role of Food Insecurity and Social Isolation

**DOI:** 10.1093/geroni/igaf122.476

**Published:** 2025-12-31

**Authors:** Pierre Jolin, Patricia Markham Risica, Julie Locher, James Jolin, Kali Thomas

**Affiliations:** Brown University, Providence, Rhode Island, United States; Brown University, Providence, Rhode Island, United States; The University of Alabama at Birmingham, Birmingham, Alabama, United States; University of Cambridge Department of Public Health and Primary Care, Cambridge, England, United Kingdom; Johns Hopkins University, Baltimore, Maryland, United States

## Abstract

As the aging U.S. population grows, so do healthcare challenges, particularly in nutrition and dietary quality. Older adults face a higher risk of malnutrition, affecting health and quality of life. Dietary intake is influenced by health, social environments, and food access, but their combined impact remains unclear among vulnerable populations. While food security is recognized as crucial for adequate nutrition, the role of social and environmental factors in dietary quality is less understood. The Deliver-EE trials aim to explore these relationships. The purpose of our investigation is to assess the relationships between social isolation, food insecurity, and dietary risk, contributing to a deeper understanding of nutrition among older adults. With a Dietary Screening Tool (DST) threshold of ≤ 60, 93% of the sample was categorized as at risk for low dietary quality. Food insecurity in the sample was 52%, yet a high proportion of both food-insecure and food-secure individuals were at risk for low dietary quality (48% and 45% respectively). No significant association was found between social isolation and DST scores. Dietary risk does not seem to be associated with food insecurity or social isolation. These findings suggest that systemic factors, rather than ability to afford food and social connectivity, drive dietary risk. Future research should examine nutritional gaps in food assistance programs and structural determinants of dietary quality in vulnerable populations.

